# Mass Cytometry Discovers Two Discrete Subsets of CD39^−^Treg Which Discriminate MGUS From Multiple Myeloma

**DOI:** 10.3389/fimmu.2019.01596

**Published:** 2019-08-02

**Authors:** Felix Marsh-Wakefield, Annabel Kruzins, Helen M. McGuire, Shihong Yang, Christian Bryant, Barbara Fazekas de St. Groth, Najah Nassif, Scott N. Byrne, John Gibson, Christina Brown, Stephen Larsen, Derek McCulloch, Richard Boyle, Georgina Clark, Douglas Joshua, Phoebe Joy Ho, Slavica Vuckovic

**Affiliations:** ^1^Discipline of Infectious Diseases and Immunology, Faculty of Medicine and Health, Central Clinical School, The University of Sydney, Sydney, NSW, Australia; ^2^Discipline of Pathology, Faculty of Medicine and Health, School of Medical Science, The University of Sydney, Sydney, NSW, Australia; ^3^School of Life Sciences, University of Technology Sydney, Sydney, NSW, Australia; ^4^Ramaciotti Facility for Human Systems Biology, The University of Sydney, Sydney, NSW, Australia; ^5^Discipline of Pathology, Sydney Medical School, The University of Sydney, Sydney, NSW, Australia; ^6^Charles Perkins Centre, The University of Sydney, Sydney, NSW, Australia; ^7^Royal Prince Alfred Hospital, Institute of Haematology, Sydney, NSW, Australia; ^8^Sydney Medical School, The University of Sydney, Sydney, NSW, Australia; ^9^Orthopaedics Department, Royal Prince Alfred Hospital, Sydney, NSW, Australia; ^10^Concord Repatriation General Hospital, ANZAC Research Institute, Concord, NSW, Australia; ^11^Faculty of Medicine, University of Queensland, Brisbane, QLD, Australia

**Keywords:** MGUS, multiple myeloma, mass cytometry, FlowSom, Treg

## Abstract

Multiple Myeloma (MM) is preceded by the clinically stable condition monoclonal gammopathy of undetermined significance (MGUS). Critical immune events that discriminate MGUS from newly diagnosed MM (ND)MM patients remain unknown, but may involve changes in the regulatory T cell (Treg) compartment that favor myeloma growth. To address this possibility, we used mass cytometry and the unsupervised clustering algorithm Flow self-organizing map (FlowSOM) to interrogate the distribution of multiple subsets within CD25^+^CD127^low/neg^Treg in matched bone marrow (BM) and peripheral blood (PB) of MGUS and NDMM patients. Both mass cytometry and flow cytometry confirmed a trend toward prevalence of CD39^−^Treg within the Treg compartment in BM and PB of NDMM patients compared to CD39^−^Treg in MGUS patients. FlowSOM clustering displayed a phenotypic organization of Treg into 25 metaclusters that confirmed Treg heterogeneity. It identified two subsets which emerged within CD39^−^Treg of NDMM patients that were negligible or absent in CD39^−^Treg of MGUS patients. One subset was found in both BM and PB which phenotypically resembled activated Treg based on CD45RO, CD49d, and CD62L expression; another subset resembled BM-resident Treg based on its tissue-resident CD69^+^CD62L^−^CD49d^−^ phenotype and restricted location within the BM. Both subsets co-expressed PD-1 and TIGIT, but PD-1 was expressed at higher levels on BM-resident Treg than on activated Treg. Within BM, both subsets had limited Perforin and Granzyme B production, whilst activated Treg in PB acquired high Perforin and Granzyme B production. In conclusion, the use of mass cytometry and FlowSOM clustering discovered two discrete subsets of CD39^−^Treg which are discordant in MGUS and NDMM patients and may be permissive of myeloma growth which warrants further study. Understanding the regulatory properties of these subsets may also advance MGUS and MM diagnosis, prognosis, and therapeutic implications for MM patients.

## Introduction

Multiple Myeloma (MM) is characterized by clonal expansion of malignant plasma cells in the bone marrow (BM). It is preceded by the clinically stable condition monoclonal gammopathy of undetermined significance (MGUS), which is defined by the presence of a monoclonal protein and <10% malignant plasma cell infiltration in the BM. The underlining immunological mechanism preventing malignant plasma cell expansion in MGUS compared to the permissive expansion of malignant plasma cells in MM patients is poorly understood and remains under active investigation (1). However, an accepted concept has been that the immune system in MM patients is tipped in favor of myeloma growth by initiating immunosuppressive mechanisms mediated by different regulatory immune cells, particularly regulatory T cells (Treg) (2).

Suppressive Treg are a subset of CD4^+^T lymphocytes driven by the expression of transcription factor forkhead box P3 (FoxP3), and play an important role in the maintenance of self-tolerance and the control of immune homeostasis (3). In humans, the Treg compartment encompasses multiple subsets which delineate different developmental stages and their associated regulatory functions (4). Based on CD45RA and FoxP3 expression Treg were divided into three subsets: resting Treg (CD45RA^+^FoxP3^lo^), activated Treg (CD45RA^−^FoxP3^hi^) and non-suppressive Treg (CD45RA^−^FoxP3^lo^) which secrete IL-10 as well as TGF-β (5). Additional phenotypic features of these defined Treg subsets include CD49d expression on activated Treg (6) and ICOS expression on IL-10 and TGF-β secreting Treg (7). In addition specific combinations of chemokine receptor expression (CCR6, CXCR3, CCR4, CCR10) can be used to define Treg subsets capable of regulating Th cell responses (8). Expression of ectonucleotidase CD39 defines a subset of Treg involved in the CD39/CD73 adenosine pathway, a key immunosuppressive mechanism operating in tumor microenvironments (9, 10).

Heterogeneity within the human Treg compartment has usually been defined by classical flow cytometry which may underestimate the complexity due to a limited multiplexing capacity as well as limitations associated with classical biaxial gating. The recent introduction of mass cytometry provides multiparametric analyses (11), combined with high-dimensional data analysis allowing the identification of 22 phenotypically distinct subsets based on 26 analyzed parameters within the Treg compartment in peripheral blood (PB) samples of healthy adults (12).

Previous studies demonstrated the ability of myeloma cells to induce activation and expansion of functional Treg in either myeloma-infiltrated BM or *in vitro* cultures (13, 14). We considered that in a growth permissive BM environment, myeloma cells may change pre-existing heterogeneity within the Treg compartment by inducing discrete Treg subsets which facilitate its progression. The emergence of these Treg subsets may support critical initiating events indicative of clinically active myeloma and potentially discriminate between MGUS and newly diagnosed (ND)MM patients. In this study we used mass cytometry and the unsupervised clustering algorithm Flow self-organizing map (FlowSOM) to interrogate at a high resolution the heterogeneity within the Treg compartment in matched BM and PB of MGUS and NDMM patients, and discovered two discrete subsets of CD39^−^Treg which are able to discriminate between these two clinical entities.

## Materials and Methods

### Patient and Healthy Donor Samples

MGUS and MM patients were recruited at the Royal Prince Alfred Hospital and diagnosed based on clinical symptoms and biopsies. Age-matched healthy donors (HD) included healthy blood donors and patients without diagnosed malignancy, autoimmune disease (including diabetes), or active infection undergoing hip arthroplasty at the Department of Orthopedic Surgery at the Royal Prince Alfred Hospital. A total of 22 patients, MGUS (*n* = 6) and NDMM (*n* = 16) and 15 HD (blood donors *n* = 6; patient *n* = 9) donated either blood, BM or both for this study. Matched BM and PB samples were always analyzed in the same experiment. Due to sampling restrictions, it was not possible to obtain matched BM and PB samples from all patients and HD or to analyze all samples by both mass cytometry and flow cytometry, so an assay selection was based on sample availability. Patient and HD characteristics and assay usage are displayed in Table S1. BM-mononuclear cells (BM-MNC) and PB-MNC were isolated by Ficoll-Hypaque (GE Healthcare) density gradient and cryopreserved prior to experimentation. The study was approved by the institutional Human Ethics Committee (X15-0357, X16-0291, X18-0096, HREC/11/CRGH/61). All patients gave written informed consent in accordance with the amended Declaration of Helsinki.

### Flow Cytometry

Antibodies used for flow cytometry analysis were: V500-CD3 (clone UCHT1, BD), APC-H7-CD8 (clone SK1, BD), V450-CD4 (clone RPA-T4, BD), PerCy5.5-CD4 (clone SK3), PE-CD25 (clone 2A3, BD), FITC-CD127 (clone HIL-7R-M21, BD), APC-CD39 (clone B249211, BioLegend), PE-Cy7-CD73 (clone AD2, BD), IgG1-APC (clone MOPC-21, BD), IgG1-PerCP-Cy5.5 (clone X40, BD), IgG1-PE-Cy7 (clone X40, BD), AF647-Foxp3 (clone 206D, Biolegend). Intranuclear FoxP3 staining was conducted using eBiosciences FoxP3 buffer kit (San Diego, CA, USA) according to the manufacturers' protocol. The LIVE/DEAD™ Fixable Violet dead cell stain kit (L34955, Invitrogen) was used to exclude dead cells. Stained cells were acquired using the BD FACSCanto II and analyzed using FlowJo software 10.4.2. Treg were defined as CD25^+^CD127^low/neg^ or CD4^+^FoxP3^+^ events.

### Mass Cytometry Staining and Data Acquisition

Three million BM-MNC or PB-MNC were stained for mass cytometry with metal-conjugated monoclonal antibodies (mAb) as listed in Table S2. Briefly, cells were stained with 1.25 μM cisplatin in RPMI for 3 min at room temperature and quenched with FACS buffer (PBS, 0.02% Sodium Azide, 0.5% BSA, and 2 mM EDTA). Cells were incubated at 4°C for 30 min initially with AF647-labeled CD160. Following a FACS wash step cells were subsequently incubated at 4°C for 30 min with a cocktail of metal-conjugated mAb targeting surface proteins. Metal-conjugated mAb were purchased from Fluidigm (Toronto, Canada) or purchased in a carrier-protein-free format and conjugated with the respective metal isotope using the MaxPAR antibody conjugation kit (Fluidigm) according to the manufacturer's recommended protocol by the Ramaciotti Facility for Human Systems Biology, Sydney, Australia. Following wash with FACS buffer, cells were fixed and permeabilized for 45 min using the eBioscience's FoxP3 buffer kit, then incubated for 30 min at 4°C with a cocktail of metal-conjugated mAb targeting intracellular proteins. Cells were subsequently washed with Perm buffer then FACS buffer, and fixed overnight in 4% paraformaldehyde containing DNA intercalator (0.125 μM iridium-191; Fluidigm). After multiple washes with FACS buffer and MilliQ water, cell concentration was adjusted to 0.8 × 10^6^ cells/mL in MilliQ water with EQ beads (Fluidigm) diluted 1 in 10 and filtered through a 35 μm nylon mesh. Cells were acquired at a rate of 200–400 cells/s using a CyTOF 2 Helios upgraded mass cytometer (Fluidigm, Toronto, Canada). Data collected in .fcs file format were normalized for signal intensity of EQ beads using the Helios software.

### Mass Cytometry Data Analysis

FlowJo 10.4.2 software (FlowJo, LLC, Ashland, OR, USA) was used to gate Treg. Our gating strategy excluded calibration beads by gating on 140 Ce^−^ events and cell aggregates using DNA signal (191Ir) and event length. CD38^+^CD3^−^ (myeloma cells), CD19^+^ (B cells), and CD56^+^ (NK cells) were excluded prior to Treg gating. Treg were identified within gated CD3^+^CD8^−^CD4^+^ T cells as CD25^+^CD127^low/neg^. All Treg with channel numbers for all markers across all samples were imported into R studio (v1.1.456). To assist in mass cytometry data analysis, the script Cytometry Analysis Pipeline for large and compleX datasets (CAPX) was utilized (15). CAPX includes both the clustering algorithm FlowSOM (16) and dimensionality reduction algorithm t-SNE (17) in a single script (https://github.sydney.edu.au/fmar5916/FMW-2019-TReg). For these algorithms, each sample contributed the same number of Treg (2,550 cells) to minimize any bias. Thirteen markers associated with Treg identification and activation were used for both clustering and dimensionality reduction. This included CD122, CD27, CD39, CD62L, CD127, CD45RA, CD69, CD49d, CD28, CD45RO, CD197, CD25, and CD38 (Table S2). FlowSOM was first run to create 25 metaclusters (MC), using two different seeds: seed A (FlowSOM_seed = 42) and seed B (FlowSOM_seed = 204) to confirm reproducibility. After this, the concatenated data were down-sampled to a total of 35,000 cells. t-SNE plots were generated on these data using the same 13 markers with the following parameters: perplexity = 30, theta = 0.5, and 1,000 iterations. Further details can be found at the CAPX script used above. Due to the limitation in patient sample availability, we were not able to accommodate all patients' samples in the computational analysis. To find a robust solution for this limitation, we ran FlowSOM and t-SNE algorithms using mass cytometry data with minimal batch fluctuation, using BM and PB of MGUS (*n* = 3) and NDMM patients (*n* = 4). Based on these results, MC of interest were further interrogated by manual gating to validate these observed changes, utilizing all available patient data from paired BM and PB of total MGUS (*n* = 4) and NDMM (*n* = 8) patients.

### Statistical Analysis

Non-parametric Mann–Whitney and Wilcoxon matched-pairs signed rank test for two samples, or Kruskal–Wallis with Dunn's multiple comparison tests for multiple samples were performed as appropriate. All statistical tests were performed at the *p* < 0.05 significance level. Statistical analyses were performed using GraphPad Prism 7 (San Diego, USA).

## Results

### Flow Cytometry Defines a Trend Toward Prevalence of CD39^−^ Cells Within the Treg Compartment of NDMM Patients

**Figure 1:**
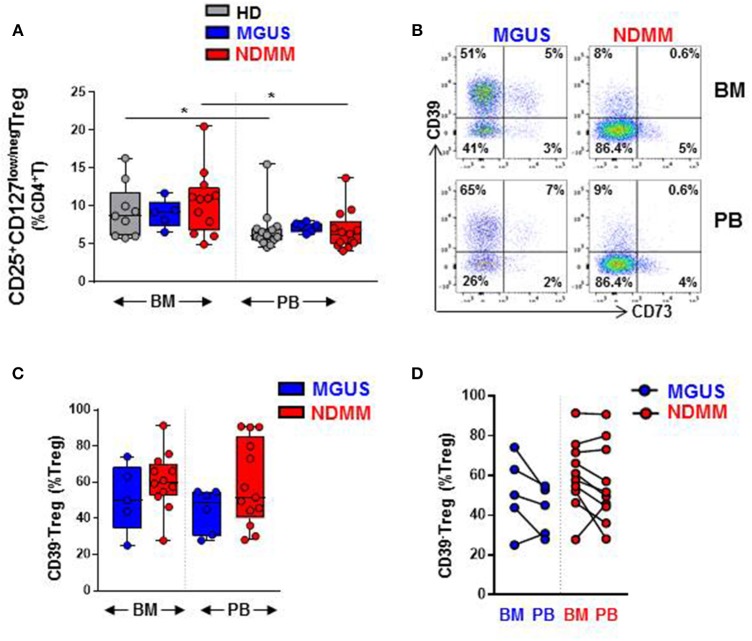
CD39^−^Treg are prevalent within the Treg compartment of NDMM patients. Flow cytometry data: **(A)** Frequency of Treg in BM and PB of HD (BM = 9, PB = 14), MGUS (BM = 5, PB = 6), and NDMM (BM = 12, PB = 13) patients. **(B)** Representative biaxial plot of CD39 vs. CD73 expression on Treg. Numbers indicate percentage of cells in each quadrant. **(C)** Frequency of CD39^−^Treg within the Treg compartment in BM and PB of MGUS (BM = 5, PB = 6) and NDMM (BM = 12, PB = 13) patients. **(D)** Frequency of CD39^−^Treg in paired BM and PB of MGUS (*n* = 5) and NDMM (*n* = 10) patients. Box and whisker plots show min and max, with median and individual data points. Multiple independent variables were analyzed using the Kruskal-Wallis test with Dunn's multiple comparison tests and 2 independent variables with the Mann-Whitney-*U*-test and Wilcoxon matched-pairs signed rank test; *p < 0.05.

Our initial analysis of Treg by flow cytometry confirmed a comparable size of CD25^+^CD127^low/neg^Treg in both BM and PB of HD, MGUS, and NDMM patients (Figure 1A). Treg represented higher proportion of CD4^+^T cells in BM then in PB of HD and NDMM patients (Figure 1A). Considering the well-established role of CD39^+^Treg in adenosine production within a suppressive tumor environment (9, 10, 18), we analyzed CD39 expression on Treg in matched BM and PB of MGUS and NDMM patients. Based on current knowledge, we expected a shift toward CD39^+^ cells within the Treg compartment of NDMM patients. In contrast to our initial hypothesis, we found a trend toward prevalence of CD39^−^Treg within both the BM and PB of NDMM compared to MGUS patients (Figures 1B,C). It appeared that CD39^−^Treg were more frequent in BM than in PB of individual MGUS and NDMM patients (Figure 1D). The increasing trend of CD39^−^Treg in NDMM patients did not affect FoxP3 expression within the Treg compartment, as Treg of NDMM and MGUS patients maintained comparable levels of FoxP3 expression (Figure S1A). We also analyzed expression of CD73 (another ectonucleotidase required for adenosine production), finding a low frequency of CD73^+^Treg across patients (Figure 1B; Figure S1B), consistent with reported low expression of CD73 on Treg in humans (19). These data suggest CD39^−^Treg although highly variable between MGUS and NDMM patients are more dominant within the Treg compartments of NDMM patients.

### Mass Cytometry Reveals That Phenotypic Organization of CD39^−^Treg Differs Between MGUS and NDMM Patients

Since flow cytometry data suggested that CD39^−^Treg can be differently represented between MGUS and NDMM patients, mass cytometry was then used to interrogate at a high resolution the phenotypic organization of the Treg compartment in BM and PB of MGUS and NDMM patients. We assembled a panel of 35 metal isotope-conjugated antibodies to simultaneously measure the expression of surface and intracellular proteins known to define stages of Treg activation, effector/suppressor function and senescence (Table S2). In non-overlapping patient cohorts, mass cytometry and flow cytometry detected a comparable frequency of Treg in the BM and PB of MGUS and NDMM patients (Figure 1A; Figure S1C).

**Figure 2:**
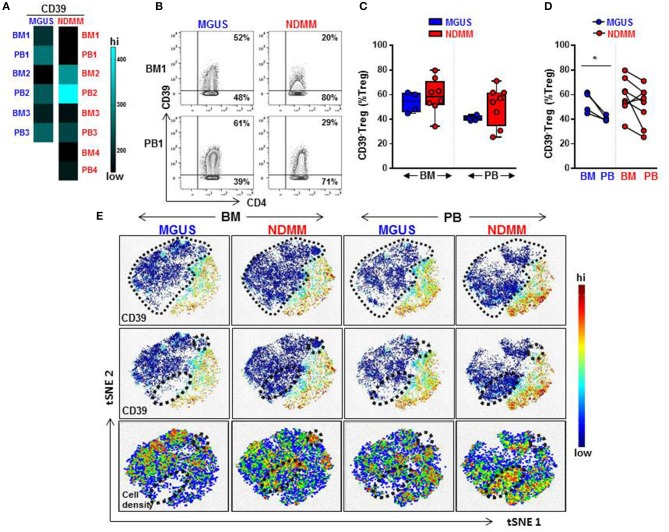
Frequency of CD39^−^Treg increases in NDMM compared to MGUS patients. Mass Cytometry data: **(A)** Heatmap of median signal intensity of CD39 expression on Treg in matched PB and BM from MGUS (*n* = 3) and NDMM (*n* = 4) patients. **(B)** Representative biaxial plots of CD39 vs. CD4 expression in the Treg compartment (CD25^+^CD127^low/neg^). Numbers indicate percentage of CD39^+^ and CD39^−^Treg. **(C)** Frequency of CD39^−^Treg in BM and PB of MGUS (*n* = 4) and NDMM (*n* = 8) patients obtained by biaxial gating shown in **(B)**. Box and whisker plots show min and max, with median and individual data points. **(D)** Frequency of CD39^−^Treg in paired BM and PB of MGUS (*n* = 4) and NDMM (*n* = 8) patients. Mann-Whitney-*U*-test and Wilcoxon matched-pairs signed rank test; *p < 0.05. **(E)** t-SNE plots of the Treg compartment in pooled BM and PB of MGUS (*n* = 3) and NDMM (*n* = 4) patients. t-SNE plots show clustering patterns of CD39^+^Treg (top, middle panels) and cell density (bottom panels). Area occupied by CD39^−^Treg (top panel) and distinct regions which are occupied differently by CD39^−^Treg (middle, bottom panels) are indicated.

Overall, a heatmap of the mass cytometry data revealed lower expression of CD39 on Treg within the BM and PB of NDMM patients compared to Treg of MGUS patients (Figure 2A). There was noticeable heterogeneity in the level of CD39 expression within NDMM patients (BM1, PB1, BM3-4, PB3-4 vs. BM2, PB2; Figure 2A). Consistent with flow cytometry data, mass cytometry confirmed the prevalence of CD39^−^Treg in both the BM and PB of NDMM patients compared to MGUS patients (Figures 2B,C). Also in line with flow cytometry data, CD39^−^Treg were more frequent in BM than in PB samples of individual MGUS and NDMM patients by mass cytometry (Figure 2D).

To assist in our phenotypic analysis of CD39^−^Treg, t-SNE plots were generated to visualize marker expression by pooling Treg from either BM or PB of MGUS and NDMM patients. As expected, based on the prevalence of CD39^−^Treg in NDMM, CD39^−^Treg occupied a larger area of Treg in NDMM patients compared to corresponding plots of MGUS patients (Figure 2E, t-SNE top panel). Interestingly, t-SNE plots displayed two distinct regions which were occupied differently by CD39^−^Treg dependent on their patient or tissue origin. In NDMM patients, CD39^−^Treg from BM occupied both, while CD39^−^Treg from PB only one of these two distinct regions. Overall, these two distinct regions occupied sparsely by CD39^−^Treg originated either from BM or PB of MGUS patients (Figure 2E, t-SNE middle panel). These CD39^−^Treg had differential cell distribution between the tissues and patients, as shown by the cell density (Figure 2E, t-SNE bottom panel). These data reveal two exciting novel points: (i) CD39^−^Treg of NDMM patients encompassed discrete subsets localized within a distinct regions of t-SNE plot which are obscured in CD39^−^Treg of MGUS patients and (ii) these discrete subsets within CD39^−^Treg are differently distributed between BM and PB of NDMM patients.

### Unsupervised FlowSOM Clustering Reveals Activated CD39^−^Treg and BM-Resident CD39^−^Treg Which Emerge in NDMM Patients

**Figure 3:**
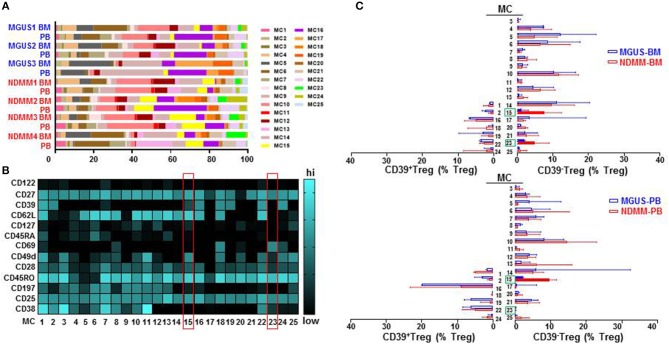
Treg compartment of MGUS and NDMM patients displayed in 25 phenotypically different MC generated by the FlowSOM. **(A)** Inter-patient heterogeneity for each sample is represented by a horizontal bar in which segment lengths represent the frequency of the Treg assigned to each MC, colored according to the accompanying legend. **(B)** Heatmap of median signal intensity of indicated surface markers (rows) and MC (columns) summarizing the 25 different phenotypes within the Treg compartment generated by FlowSOM (seed A). The phenotypes of Treg in MC15 and MC23 are indicated on the heatmap (red rectangles). **(C)** Horizontal bars are frequency of Treg in each MC. MC occupied by CD39^+^Treg (left) and by CD39^−^Treg (right); MC15 and MC23 are indicated (closed bars, green rectangles). Bars are medians with interquartile range.

To interrogate the phenotypic organization of the Treg compartment and further define the phenotype of two discrete subsets of CD39^−^Treg which emerged in NDMM patients, we performed unsupervised clustering analysis of mass cytometry data using FlowSOM (16). FlowSOM was performed using the same number of CD25^+^CD127^low/neg^Treg from each BM and PB samples of MGUS (*n* = 3) and NDMM (*n* = 4) patients and 13 parameters (Table S2) associated with Treg identification and activation (4). We decided to split the Treg compartment into 25 phenotypically different MC (Figure 3), based on the recently reported number of 22 MC using 26 parameters in the Treg compartment in PB of HD (12). To evaluate the robustness of these MC, we used two different FlowSOM seeds (seed A, B) and observed high reproducibility in term of MC size and phenotype (Figure 3; Figure S2). We evaluated each patient sample quantitatively in term of its MC distribution and demonstrated each sample was unique in terms of the Treg frequency assigned to each MC (Figure 3A; Figure S2A), reflecting inter-patient differences within and between MGUS and NDMM cohorts.

**Figure 4:**
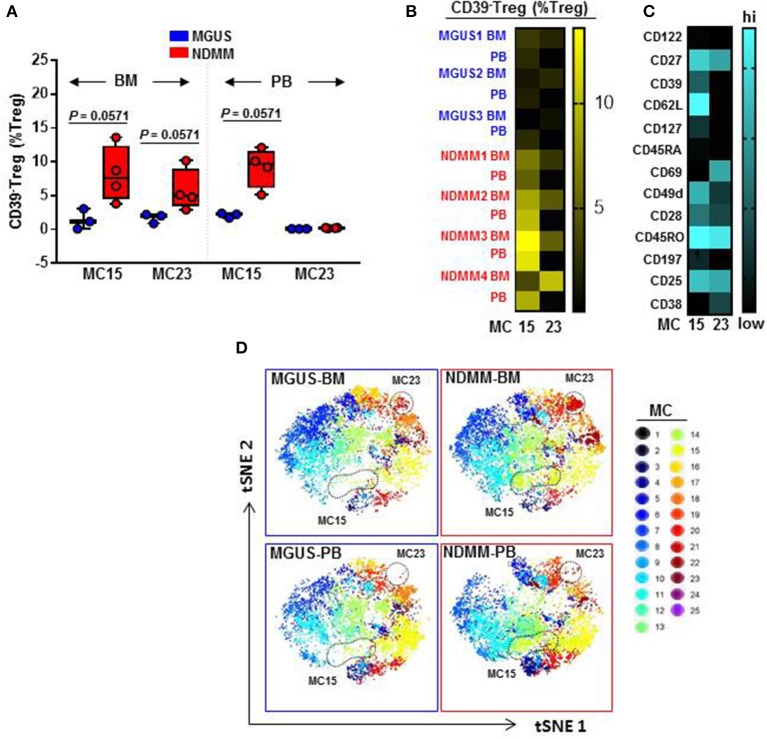
Activated CD39^−^Treg and BM-resident CD39^−^Treg emerge in NDMM patients. **(A)** Frequency of Treg in MC15 and MC23 in BM and PB of MGUS (*n* = 3) and NDMM (*n* = 4) patients. Box and whisker plots show min and max, with median and individual data points. **(B)** Heatmap of Treg frequency in MC15 and MC23 in individual BM and PB from MGUS (*n* = 3) and NDMM patients (*n* = 4). **(C)** Phenotype of Treg in MC15 and MC23. **(D)** t-SNE plots display MC in pooled BM and PB of MGUS (*n* = 3) and NDMM (*n* = 4) patients colored according to the accompanying legend. MC15 and MC23 are indicated by dotted lines.

We found that CD39^−^Treg encompassed a majority of MC, and were more heterogeneous compared to their CD39^+^Treg counterparts (18 vs. 7 MC, respectively, Figures 3B,C; Figures S2B,C). MC occupied by CD39^+^Treg were consistently present across tissues and patients (Figure 3C; Figure S2C), suggesting that tissue residency or patient specificity was not embedded to CD39^+^Treg. Among all MC displaying CD39^−^Treg in BM and PB of MGUS and NDMM patients, the occupancy of MC15 and MC23 appeared to be consistently different between patients and tissues (Figures 4A,B). CD39^−^Treg in MC15 represented 7% and 9.7% of Treg in BM and PB, respectively of NDMM patients. CD39^−^Treg in MC23 was found exclusively in the BM of NDMM patients and represented 5% of Treg. In MGUS patients, both MC15 and MC23 were almost undetectable (Figures 4A,B). MC15 phenotypically resembled activated Treg based on CD45RO, CD49d, and CD62L expression (Figure 4C). Interestingly, MC23 had a core signature defining tissue residency, including CD69 expression, a lack of CD62L and CD49d expression and exclusive detection in the BM (Figures 4A–C).

To assist in data visualization, t-SNE plots were generated showing MC location. These t-SNE plots revealed that MC15 and MC23 overlapped with two previously defined regions within the CD39^−^Treg that differed between patients and tissues (Figures 2E, 4D). Two different FlowSOM seeds produced similar results (Seed A in Figures 3, 4; Seed B in Figures S2, S3). This concludes that two discrete subsets defined as activated CD39^−^Treg and BM-resident CD39^−^Treg emerge in NDMM and discriminate MGUS from clinically active myeloma.

### Activated CD39^−^Treg and BM-Resident CD39^−^Treg Can Be Matched by Classical Biaxial Gating of Mass Cytometry Data

**Figure 5:**
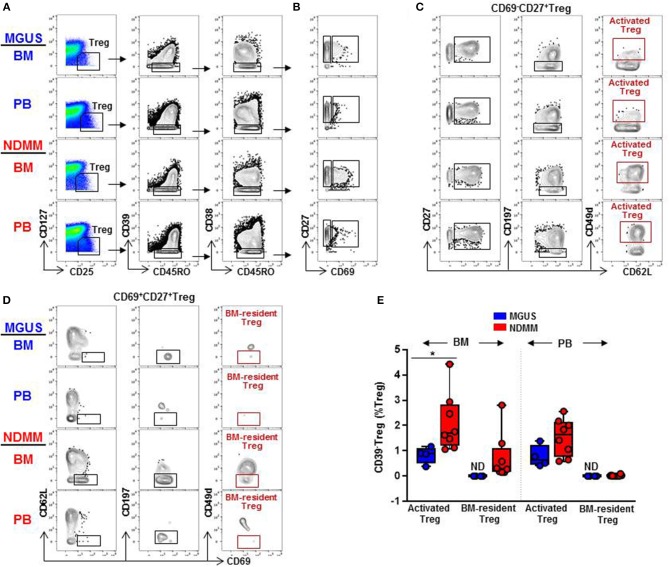
Biaxial gating of mass cytometry data to match activated CD39^−^Treg (MC15) and BM-resident CD39^−^Treg (MC23) in MGUS and NDMM samples. **(A)** Representative biaxial gating of CD45RO^+^CD39^−^CD38^−^Treg displayed in a plot of CD27 vs. CD69 in **(B)**. Representative gating strategy to match **(C)** activated CD39^−^Treg (MC15) within CD69^−^CD27^+^Treg and **(D)** BM-resident CD39^−^Treg (MC23) within CD69^+^CD27^+^Treg. **(E)** Frequency of activated CD39^−^Treg and BM-resident CD39^−^Treg in matched BM and PB samples of MGUS (*n* = 4) and NDMM (*n* = 8) patients. *p < 0.05. ND, not defined.

Distinct clusters produced by computational clustering of mass cytometry data have to be matched in multiple samples to demonstrate their biological reality. Therefore, we next analyzed whether activated CD39^−^Treg and BM-resident CD39^−^Treg defined by FlowSOM clustering can be matched, quantified, and compared between samples by classical biaxial gating of mass cytometry data. We developed a biaxial gating strategy to fit the MC phenotype defined by automated FlowSOM clustering, and applied this same gating strategy to BM and PB samples of MGUS (*n* = 4) and NDMM (*n* = 8) patients (Figure 5). It is worth noting that one of the total 4 MGUS and 4 of the total 8 NDMM patients did not contribute to the MC phenotypes defined in the original FlowSOM clustering (Figure 3B). However, by classical biaxial gating activated CD39^−^Treg and BM-resident CD39^−^Treg were well-separated from the other CD45RO^+^CD38^−^Treg (Figures 5A,B). Activated CD39^−^Treg phenotypically resembled MC15 defined by gating on CD27^+^CD69^−^CD62L^+^CD197^−^CD49d^+^Treg(Figures 5B,C). BM-resident CD39^−^Treg phenotypicallyresembled MC23 defined by gating on CD27^+^CD69^+^CD62L^−^CD197^−^CD49d^−^Treg (Figures 5B,D). Although frequency of activated CD39^−^Treg and BM-resident CD39^−^Treg were generally smaller by biaxial gating than by FlowSOM clustering, these subsets were prevalent in NDMM and obscured or absent in MGUS samples by biaxial gating (Figure 5E). This proves biaxial gating analysis effective in matching activated CD39^−^Treg and BM-resident CD39^−^Treg, and overlap between the biaxial gating and the clustered analysis for these Treg subsets.

### Both CD39^−^Treg Subsets Co-expressed PD-1 and TIGIT But PD-1 Was Expressed at Higher Levels on BM-Resident Treg Then on Activated Treg

**Figure 6:**
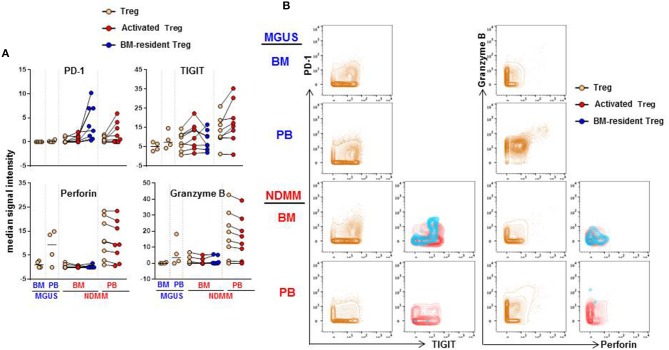
Expression of PD-1, TIGIT, Perforin and Granzyme B on activated CD39^−^Treg and BM-resident CD39^−^Treg **(A)**. Median signal intensity of PD-1, TIGIT, Perforin. and Granzyme B defined by biaxial gating in total Treg, activated CD39^−^Treg and BM-resident CD39^−^Treg in BM and PB of MGUS (*n* = 4) and NDMM (*n* = 8) patients. Total Treg, activated CD39^−^Treg and BM-resident CD39^−^Treg from individual NDMM patients were connected. **(B)** Representative biaxial plots of PD-1 vs. TIGIT and Granzyme B vs. Perforin expression in total Treg, activated CD39^−^Treg and BM-resident CD39^−^Treg in matched BM and PB of individual MGUS and NDMM patient.

We considered that activated CD39^−^Treg and BM-resident CD39^−^Treg which develop in the stage of active disease in NDMM patients may acquire inhibitory check point molecules, or markers of effector/senescent cells, that are associated with enhanced Treg-mediated suppression in the tumor environment (20). Thus, we compared expression levels of 13 markers on total Treg, activated CD39^−^Treg and BM-resident CD39^−^Treg in MGUS and NDMM patients by biaxial gating (Figure 6, data not shown). PD-1 and TIGIT were expressed at higher levels on activated CD39^−^Treg in both BM and PB then on total Treg population. In the majority of patients, PD-1 and TIGIT expression was comparable on activated CD39^−^Treg in the BM and PB (Figure 6A). PD-1 was expressed at greater levels but TIGIT was at lower levels on BM-resident Treg then on activated CD39^−^Treg (Figures 6A,B). Both CD39^−^Treg subsets co-expressed PD-1 and TIGIT (Figure 6B). Other inhibitory checkpoint molecules PD-L1, Lag3, Tim3, CD160, CXCR3 [a chemokine receptor associated with effector/memory cells (21)], KLRG1 [a marker of replicative senescence (22)], marker of proliferation Ki67, transcription factors Tbet and Eomes [regulators of T cell-mediated cytotoxicity (23)] had low-intensity staining undistinguishable from background (data not shown), consistent with reported low expression of these molecules on human Treg (24). Despite the lack of Tbet and Eomes expression, both activated CD39^−^Treg and BM-resident CD39^−^Treg produced Perforin and Granzyme B at levels similar to the total Treg population. There was an obvious difference in Perforin and Granzyme B production by Treg in BM and PB, such as Treg in PB (including activated CD39^−^Treg) were major producers of both Perforin and Granzyme B (Figures 6A,B). There was noticeable inter-patient heterogeneity in the levels of Perforin and Granzyme B expression by total Treg within MGUS and NDMM cohorts, thus more data are required to interpret Perforin and Granzyme B expression by Treg across patient cohorts with confidence. Nevertheless, these data revealed important novel attributes associated with Treg in NDMM patients, including co-expression of PD-1 and TIGIT on both Treg subsets and higher levels of PD-1 expression on BM-resident CD39^−^Treg than on activated Treg.

## Discussion

One of the challenges in the field of plasma cell dyscrasia and myeloma is to understand which factors keep MGUS clinically stable, and what critical events allow permissive expansion of malignant plasma cells to lead to the progression of clinical MM. This is particularly perplexing as MGUS patients have already been shown to have increased infiltration of T cells within the BM, as well as frequent TCR-Vβ expansions indicative of ongoing immune responses, and genetic changes similar to MM patients (25–28). Here, we provide an exciting novel discovery which may help to resolve this mystery. We discovered that two discrete subsets: activated CD39^−^Treg and BM-resident CD39^−^Treg emerge in NDMM, thus allowing discrimination between MGUS and NDMM patients. The availability and use of mass cytometry which allows interrogation of the Treg compartment of MGUS and NDMM patients at high resolution has facilitated the discovery of these two discrete subsets of CD39^−^Treg.

Treg have been implicated in myeloma progression based on their contribution to the complex immunosuppressive environment through secretion of cytokines IL-10 and TGF-β (29), as well as direct inhibition of effector T cell responses (30). An additional important suppressive mechanism mediated by Treg involves the CD39/CD73 adenosine pathway. In this pathway, Treg expressing the ectonucleotidase CD39 in conjunction with CD73-expressing cells hydrolyse extracellular ATP and generate adenosine. Extracellularly produced adenosine following the engagement with its cognate receptors suppress effector T cell responses and induces myeloid-derived suppressor cells (18). Besides its immunosuppressive function, adenosine is also a growth factor for osteoblasts and osteoclasts (31), further implicating the importance of the CD39/CD73 adenosine pathway in MM pathogenesis. Expression of CD39 is upregulated on several solid tumors (including colorectal, pancreatic, head and neck cancer), implicating the CD39/CD73 adenosine pathway in the pathogenesis of a number of malignancies (32).

Based on all preceding observations, it is reasonable to expect that CD39^+^Treg can be maintained or even increased in malignant MM when compared to the premalignant MGUS setting. In humans, CD39^+^Treg are defined as activated effector memory cells (33) and are implicated in the suppression of Th17 responses and the control of autoimmunity (34). Unexpectedly, we found a trend toward prevalence of CD39^−^Treg along with preserved FoxP3 expression within the Treg compartment of NDMM patients. CD39^−^Treg with a preserved suppressor function pertinent to Treg have been reported as crucial effector/pathogenic cells that produce IL-17 in patients with multiple sclerosis (34). A role for CD39^−^Treg in MM has never been described before, but it may be speculated that these cells can serve as IL-17-producing myeloma-promoting cells, particularly in the myeloma-permissive BM environment (35, 36). It is interesting that both IL-17 and now the prevalence of CD39^−^Treg are common in the pathology of MM and autoimmunity. We observed noticeable heterogeneity between NDMM patients in the frequency of CD39^−^Treg. The inclusion of increased numbers of patients in future studies than presented in this preliminary study will support our observation and establishment of potential dissimilarity between CD39^−^Treg in MGUS and NDMM patients.

Previous studies have reported the production of IL-17 and IL-10 by a small proportion of CD39^−^Treg (34), however the phenotype and function of the remaining major portion of CD39^−^Treg remains uncertain. Using the advantages afforded by mass cytometry, we interrogated at a high resolution the phenotypic organization of CD39^−^Treg in matched BM and PB of MGUS and NDMM patients. From our knowledge, this report represents the first description of a phenotypic organization of CD39^−^Treg that provides some novel and exciting findings relevant to MGUS and NDMM patients. We found that CD39^−^Treg in both MGUS and NDMM patients encompass multiple MC, suggesting that they are undergoing an intensive continuum of differentiation that involves multiple stages. In contrast, their CD39^+^Treg counterpart appeared to be less dynamic and encompass a limited number of MC. We discovered subsets of activated CD39^−^Treg and BM-resident CD39^−^Treg that emerge in NDMM, and are thus able to discriminate between MGUS and NDMM patients. These two subsets are likely resistant to anti-CD38 monoclonal antibody therapy (37) based on their low/lack of CD38 expression. Based on their TIGIT and PD-1 co-expression, they may represent recently activated Treg. Remarkably, BM-resident CD39^−^Treg have higher PD-1 expression than activated Treg and may represent exhausted Treg with the highest suppressive activity (38) induced in myeloma-infiltrated BM.

Our data suggest the compelling possibility that MGUS and NDMM patients can be discriminated based on the presence of activated CD39^−^Treg and BM-resident CD39^−^Treg. These Treg subsets differently presented between MGUS and NDMM patients initially revealed by automated FlowSOM clustering and subsequently matched and quantified in an expanded panel of MGUS and NDMM patients by classical biaxial gating of mass cytometry data. Thus, activated CD39^−^Treg and BM-resident CD39^−^Treg represent distinct biologically-relevant cell types and as such need to be matched, quantified and compared in a larger cohort of patients in different phases of disease.

Our data also raises several outstanding questions which will inspire further studies. Does myeloma directly or indirectly (through changes to myeloma-infiltrated BM) induce activated CD39^−^Treg and BM-resident CD39^−^Treg? What is the ontological origin of these two subsets of CD39^−^Treg? Are they a result of CD39 downregulation, precursors of CD39^+^Treg cells or are they developmentally independent of CD39^+^Treg? Are they clonal Treg with myeloma antigen specificity? Do they relate to the currently established categories of human Treg, or do they represent a novel type of Treg? Are BM-resident CD39^−^Treg responsible for maintenance of the myeloma niches and how do they relate to Treg being implicated in the maintenance of the haematopoietic stem cell and plasma cell niches (39, 40)?

Emergence of activated CD39^−^Treg and BM-resident CD39^−^Treg may represent necessary early changes in normal physiological Treg biology adopted by malignant myeloma cells to allow progression from MGUS to clinical MM. These changes in the Treg compartment of NDMM patients have real potential to improve our understanding of the clinical stability in MGUS and disease progression into MM, to further advance clinical diagnosis, prognosis, and therapeutic implications for MM.

## Data Availability

The raw data supporting the conclusions of this manuscript will be made available by the authors, without undue reservation, to any qualified researcher.

## Ethics Statement

This study was carried out in accordance with the recommendation of Royal Prince Alfred Hospital (X15-0357, X16-0291, X18-0096) and ANZAC Research Institute (HREC/11/CRGH/61) Ethics committee. All subjects gave written informed consent in accordance with the Declaration of Helsinki.

## Author Contributions

FM-W and AK performed the research, analyzed the data, and wrote the paper. HM designed and performed mass cytometry assays and wrote the paper. SY performed the research and analyzed the data. CBry reviewed patients, wrote the Human Ethics and analyzed the data. BF designed research and assisted in mass cytometry assays. NN, DJ, and PH designed research and wrote the paper. SB assisted Flow/CAPX approach, analyzed FLowSOM data and wrote the paper. JG, CBro, SL, and DM reviewed patients and designed research. RB reviewed patients undergoing hip arthroplasty and designed research. GC assisted collection of the healthy donor samples, designed research, analyzed data. SV designed research, performed experiments, analyzed data, and wrote the paper.

### Conflict of Interest Statement

The authors declare that the research was conducted in the absence of any commercial or financial relationships that could be construed as a potential conflict of interest.

## Supplementary Material

Figure S1 Treg detection by flow cytometry and mass cytometry in the BM and PB of MGUS and NDMM patients. **(A,B)** Treg detection by flow cytometry: **(A)** Representative biaxial plot of Treg defined as FoxP3^+^ events. Numbers indicate percentage of FoxP3^+^Treg. Representative histograms of the FoxP3 intensity in the Treg compartment in matched BM and PB of MGUS (*n* = 2) and NDMM (*n* = 3) patients. **(B)** Representative biaxial plot of Treg defined as CD25^+^CD127^low/neg^. Numbers indicate percentage of CD25^+^CD127^low/neg^ Treg. Frequency of CD73^+^Treg within the Treg compartment in BM and PB of MGUS (BM = 5, PB = 6) and NDMM (BM = 12, PB = 13) patients. **(C)** Representative biaxial plot of Treg defined as CD25+CD127^low/neg^ by mass cytometry. Numbers indicate percentage of CD25^+^CD127^low/neg^ Treg. Frequency of Treg in BM and PB of MGUS (*n* = 4) and NDMM (*n* = 8) patients. Box and whisker plots show min and max, with median and individual data points.

Figure S2 Treg compartment of MGUS and NDMM patients displayed in 25 phenotypically different MC generated by the FlowSOM (seed B). Data presented as in Figure 3 **(A–C)** (FlowSom seed A) demonstrating reproducibility in terms of MC size and phenotype across seed changes (Seed A vs. seed B). MC15 and MC23 depicted in Seed A (Figure 3) correspond to MC21 and MC1 depicted in Seed B, respectively.

Figure S3 Activated CD39^−^Treg and BM-resident CD39^−^Treg emerge in NDMM patients (seed B). Data presented as in Figure 4 **(A–D)** (FlowSom seed A) demonstrating reproducibility in terms of MC size and phenotype across seed changes (Seed A vs. seed B). MC15 and MC23 depicted in Seed A (Figure 4) correspond to MC21 and MC1 depicted in Seed B, respectively.

Table S1 Patients and HD characteristics and assay used for each subject included in study.

Table S2 List of antibodies, clones, and conjugates used for mass cytometry staining and data analysis.
