# Supplementary figures and images for: Mass Cytometry Discovers Two Discrete Subsets of CD39−Treg Which Discriminate MGUS From Multiple Myeloma
Source: Front Immunol. 2019 Aug 2;10:1596. doi: 10.3389/fimmu.2019.01596 (PMC6688400; doi:10.3389/fimmu.2019.01596)

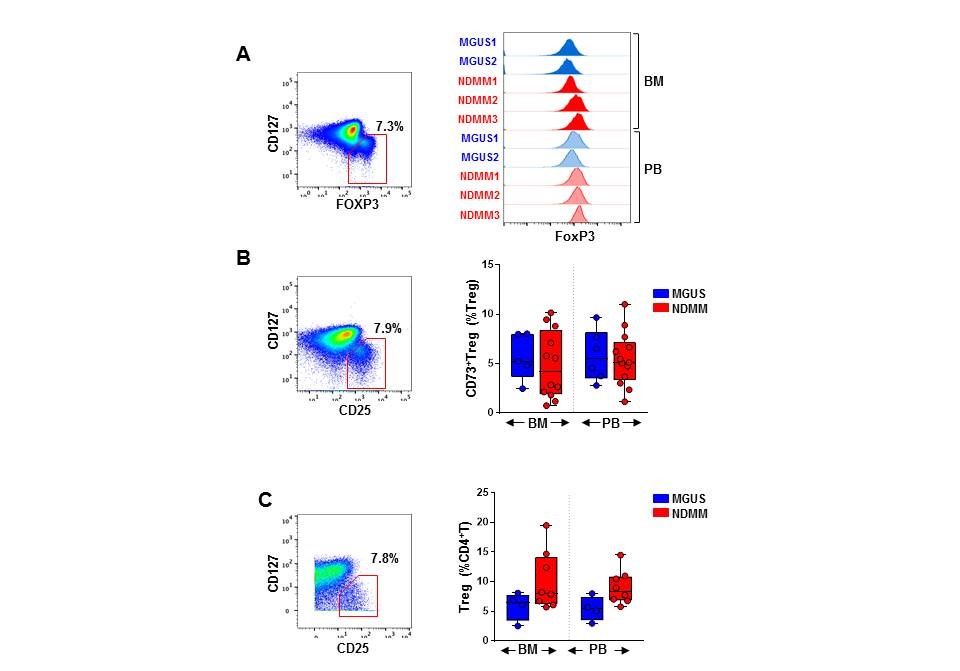

Supplement: Figure S1 — Treg detection by flow cytometry and mass cytometry in the BM and PB of MGUS and NDMM patients. (A,B) Treg detection by flow cytometry: (A) Representative biaxial plot of Treg defined as FoxP3+ events. Numbers indicate percentage of FoxP3+Treg. Representative histograms of the FoxP3 intensity in the Treg compartment in matched BM and PB of MGUS (n = 2) and NDMM (n = 3) patients. (B) Representative biaxial plot of Treg defined as CD25+CD127low/neg. Numbers indicate percentage of CD25+CD127low/neg Treg. Frequency of CD73+Treg within the Treg compartment in BM and PB of MGUS (BM = 5, PB = 6) and NDMM (BM = 12, PB = 13) patients. (C) Representative biaxial plot of Treg defined as CD25+CD127low/neg by mass cytometry. Numbers indicate percentage of CD25+CD127low/neg Treg. Frequency of Treg in BM and PB of MGUS (n = 4) and NDMM (n = 8) patients. Box and whisker plots show min and max, with median and individual data points. [file Image_1.jpeg]

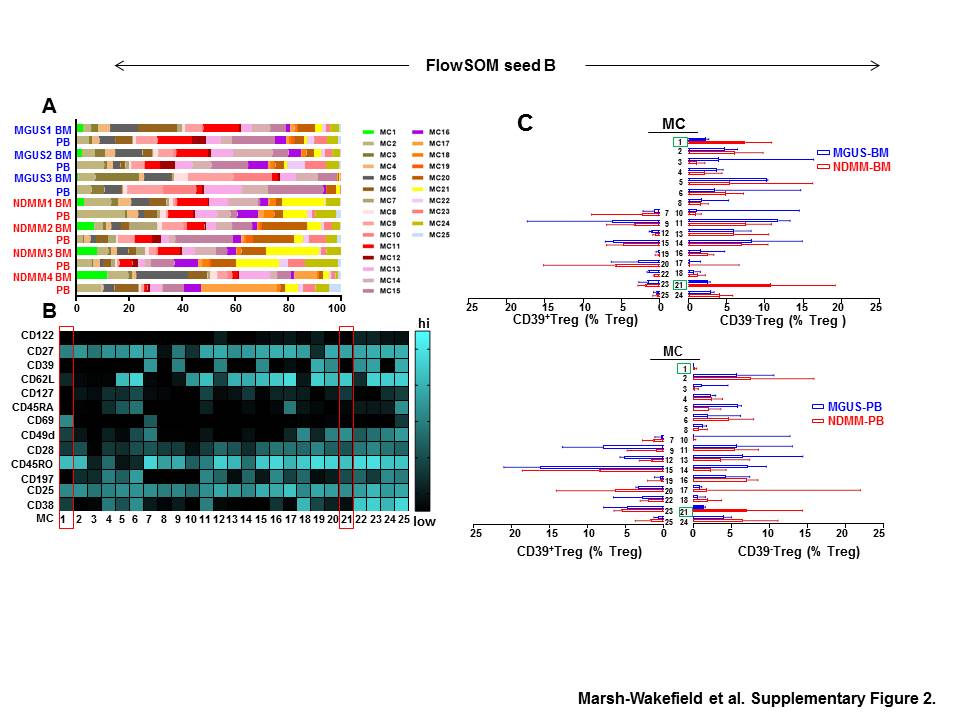

Supplement: Figure S2 — Treg compartment of MGUS and NDMM patients displayed in 25 phenotypically different MC generated by the FlowSOM (seed B). Data presented as in Figure 3 (A–C) (FlowSom seed A) demonstrating reproducibility in terms of MC size and phenotype across seed changes (Seed A vs. seed B). MC15 and MC23 depicted in Seed A (Figure 3) correspond to MC21 and MC1 depicted in Seed B, respectively. [file Image_2.JPEG]

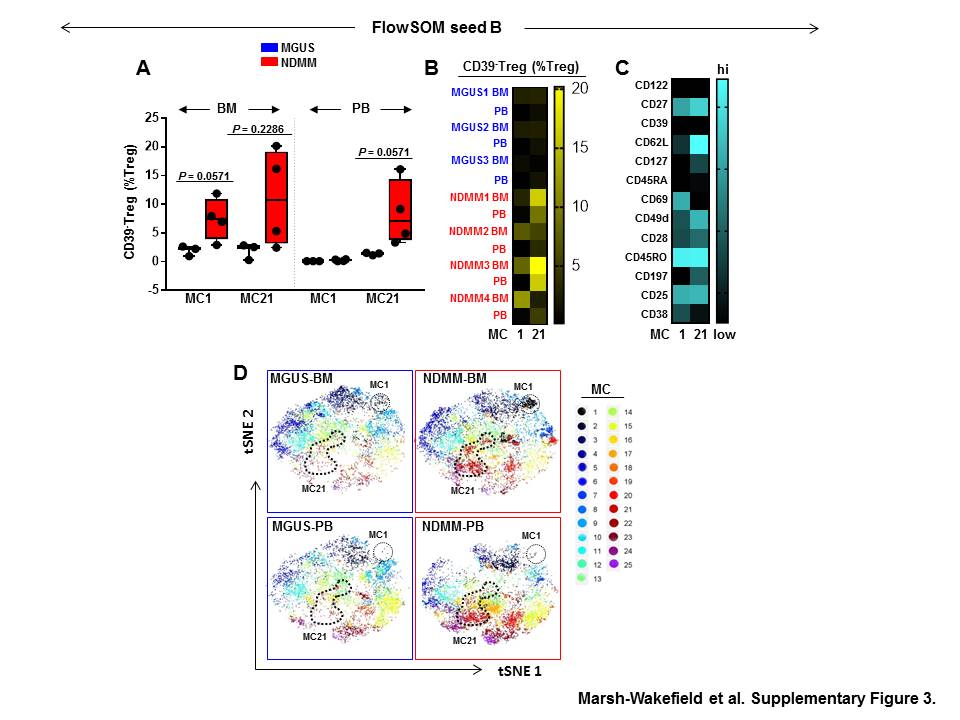

Supplement: Figure S3 — Activated CD39−Treg and BM-resident CD39−Treg emerge in NDMM patients (seed B). Data presented as in Figure 4 (A–D) (FlowSom seed A) demonstrating reproducibility in terms of MC size and phenotype across seed changes (Seed A vs. seed B). MC15 and MC23 depicted in Seed A (Figure 4) correspond to MC21 and MC1 depicted in Seed B, respectively. [file Image_3.JPEG]
