# Supplementary material for: Mass Cytometry Discovers Two Discrete Subsets of CD39−Treg Which Discriminate MGUS From Multiple Myeloma
Source: Front Immunol. 2019 Aug 2;10:1596. doi: 10.3389/fimmu.2019.01596 (PMC6688400; doi:10.3389/fimmu.2019.01596)
Supplement: Table S1 — Patients and HD characteristics and assay used for each subject included in study. [file Data_Sheet_1.PDF]

**Table S1.** Patients and HD characteristics and assay used for each subject included in study.

| Subjects | Age (years) | Sex | ISS | Sample | Analysis |
|---|---|---|---|---|---|
| MGUS1 | 64 | M | | BM/PB | Flow/Mass |
| MGUS2 | 58 | M | | BM/PB | Flow/Mass |
| MGUS3 | 56 | F | | BM/PB | Flow/Mass |
| MGUS4 | 70 | F | | NA/PB | Flow |
| MGUS5 | 70 | F | | BM/PB | Flow |
| MGUS6 | 65 | F | | BM/PB | Flow/Mass |
| | | | | | |
| NDMM1 | 83 | M | II | BM/PB | Flow/Mass |
| NDMM2 | 76 | M | I | NA/PB | Flow |
| NDMM3 | 62 | F | I | NA/PB | Flow |
| NDMM4 | 68 | F | NA | BM/PB | Flow |
| NDMM5 | 90 | M | II | BM/PB | Flow/Mass |
| NDMM6 | 65 | M | III | BM/PB | Flow |
| NDMM7 | 73 | F | II | BM/PB | Flow/Mass |
| NDMM8 | 68 | F | III | BM/NA | Flow/Mass |
| NDMM9 | 69 | M | II | NA/PB | Flow/Mass |
| NDMM10 | 39 | M | I | BM/PB | Flow/Mass |
| NDMM11 | 44 | M | I | BM/PB | Flow/Mass |
| NDMM12 | 63 | M | II | BM/PB | Flow |
| NDMM13 | 55 | F | I | BM/PB | Flow |
| NDMM14 | 53 | M | III | BM/NA | Flow |
| NDMM15 | 39 | F | III | BM/NA | Flow |
| NDMM16 | 63 | M | III | BM/PB | Flow/Mass |
| | | | | | |
| [#]HD1 | 59 | F | | BM/PB | Flow |
| [#]HD2 | 68 | F | | BM/PB | Flow |
| [#]HD3 | 71 | M | | BM/PB | Flow |
| [#]HD4 | 77 | F | | BM/PB | Flow |
| [#]HD5 | 70 | M | | BM/PB | Flow |
| [#]HD6 | 70 | M | | BM/PB | Flow |
| [#]HD7 | 66 | M | | BM/NA | Flow |
| [#]HD8 | 58 | M | | BM/PB | Flow |
| [#]HD9 | 83 | F | | BM/PB | Flow |
| [&]HD10 | 35 | F | | NA/PB | Flow |
| [&]HD11 | 60 | M | | NA/PB | Flow |
| [&]HD12 | 56 | M | | NA/PB | Flow |
| [&]HD13 | 49 | F | | NA/PB | Flow |
| [&]HD14 | 62 | F | | NA/PB | Flow |
| [&]HD15 | 59 | F | | NA/PB | Flow |

MGUS, monoclonal gammopathy of undetermined significance; NDMM, , newly diagnosed multiple myeloma; HD, healthy donor; BM, bone marrow; PB, peripheral blood; ISS, Multiple Myeloma International Staging System; NA, not available. [#]patients undergoing hip arthroplasty; [&]healthy blood donors.
