# Supplementary material for: Mass Cytometry Discovers Two Discrete Subsets of CD39−Treg Which Discriminate MGUS From Multiple Myeloma
Source: Front Immunol. 2019 Aug 2;10:1596. doi: 10.3389/fimmu.2019.01596 (PMC6688400; doi:10.3389/fimmu.2019.01596)
Supplement: Table S2 — List of antibodies, clones, and conjugates used for mass cytometry staining and data analysis. [file Data_Sheet_2.PDF]

Table S2. List of antibodies, clones and conjugates used for mass cytometry staining and data analysis

Marker	Clone	Conjugate	Company
CD160-AF647	BY55		BD
CD45	HI30	110Pd	BD
CD56	REA196	113In	Miltenyi
CD8	RPA-T8	115In	Biolegend
CD57	HCD57	139La	Biolegend
&CD49d	9F10	141Pr	Fluidigm
CD19	HIB19	142Ce	BD
&CD45RA	HI100	143Nd	Biolegend
&CD69	FN50	144Nd	Biolegend
CD4	RPA-T4	145Nd	BD
EOMES	WD1928	146Nd	Life Technologies
Cy5 (for AF647 detection)	CY5-15	147Sm	Sigma
&CD28	CD28.2	148Sm	Biolegend
CD366 (Tim3)	7D3	149Sm	BD
KLRG1	SA231A2	150Sm	Biolegend
&CD39	A1	151Eu	Biolegend
&CD45RO	UCHL1	152Sm	BD
&CD62L	DREG-56	153Eu	Biolegend
CD137	4B4-1	155Gd	BD
CD279 (PD-1)	EH12.2H7	156Gd	Biolegend
&CD197 (CCR7)	150503	159Tb	R&D Systems
CD223 (Lag3)	17B4	160Dy	Novus Biologicals
&CD122 (IL-2RB)	TU27	161Dy	Biolegend
CD183 (CXCR3)	REA232	163Dy	Miltenyi
CD274 (PDL1)	MIH1	164Dy	BD
TIGIT	MBSA43	166Er	eBioscience
&CD27	M-T271	167Er	Biolegend
Ki67	B56	168Er	BD
&CD25	M-A251	169Tm	Biolegend
CD3	UCHT1	170Yb	BD
Granzyme B	GB11	171Yb	Acris
&CD38	HIT2	172Yb	Fluidigm
Integrin ß7	FIB504	173Yb	BD
Perforin	B-D48	175Lu	Fluidigm
&CD127	A019D5	176Lu	Biolegend
T-bet	4B10	209Bi	BD

&Marker used for FlowSOM clustering and t-SNE plots.
